# Determination of the Relative Potency of Norepinephrine and Phenylephrine Given as Infusions for Preventing Hypotension During Combined Spinal-Epidural Anesthesia for Cesarean Delivery: A Randomized Up-And-Down Sequential Allocation Study

**DOI:** 10.3389/fphar.2022.942005

**Published:** 2022-07-14

**Authors:** Jing Qian, Yan-Ping Zhao, Jia-Li Deng, Li-Zhong Wang, Fei Xiao, Bei Shen, Han-Qing Yao

**Affiliations:** ^1^ Department of Anesthesia, Jiaxing Women and Children’s Hospital of Wenzhou Medical University, Jiaxing, China; ^2^ Jiaxing University Affiliated Women and Children Hospital, Jiaxing, China

**Keywords:** spinal, anaesthesia, cesarean section, norepinephrine, phenylephrine, infusions, intravenous

## Abstract

**Purpose:** The relative potency of norepinephrine and phenylephrine given as boluses to treat hypotension during spinal anesthesia for cesarean delivery has been reported but few data are available for infusions. This study aimed to determine the relative potency of norepinephrine and phenylephrine when given by infusion for preventing hypotension during combined spinal-epidural anesthesia for cesarean delivery.

**Methods:** This was a prospective, randomized, double-blind, up-and-down sequential allocation study. Patients were randomly allocated to receive a prophylactic infusion of norepinephrine or phenylephrine started immediately after induction of anesthesia. The first patients received either norepinephrine 0.1 μg/kg/min or phenylephrine 0.5 μg/kg/min. An effective infusion rate was defined when no hypotension occurred before delivery. For each subsequent patient, the norepinephrine infusion rate was decreased or increased by 0.01 μg/kg/min or the phenylephrine infusion rate was decreased or increased by 0.05 μg/kg/min according to whether the infusion was effective or ineffective respectively in the previous patient. Values for the infusion rate that was effective in preventing hypotension in 50% of patients (ED50) for norepinephrine and phenylephrine were estimated using up-and-down sequential analysis and relative potency was estimated. Probit regression was used as a backup and sensitivity analysis.

**Results:** The ED50 values for norepinephrine and phenylephrine calculated by the up-and-down method were 0.061 (95% CI 0.054–0.068) μg/kg/min and 0.368 (95% CI 0.343–0.393) μg/kg/min respectively. The estimated relative potency ratio for ED50 for norepinephrine to phenylephrine was 6.03:1 (95% CI 5.26:1 to 6.98:1).

**Conclusion:** Under the conditions of this study, norepinephrine given by infusion was about 6 times more potent than phenylephrine. This information is useful for clinical practice and further comparative studies of norepinephrine versus phenylephrine.

**Clinical Trial Registration:** http://www.chictr.org.cn/showproj.aspx, identifier [ChiCTR2200056237]

## Introduction

Phenylephrine is regarded as the first-line vasopressor for treating and preventing hypotension during spinal and combined spinal-epidural (CSE) anesthesia for cesarean delivery (Ngan Kee, 2017a; Kinsella et al., 2018). However, phenylephrine may cause reflex bradycardia which may result in a decrease in cardiac output (Ngan Kee et al., 2015a; Singh et al., 2020). These concerns have led to investigation of norepinephrine as an alternative vasopressor (Ngan Kee et al., 2015a; Ngan Kee et al., 2020a). When comparing norepinephrine and phenylephrine, for both clinical and research purposes it is useful to know their relative potency. Previously, estimates of the latter have been reported when the drugs were given by bolus (Ngan Kee, 2017b; Mohta et al., 2019). However, few data are available on the relative dose requirements of these vasopressors when they are given by infusion. This is important because infusions are a common and recommended method in clinical practice (Kinsella et al., 2018; Doherty et al., 2012; das Neves et al., 2010) and differences in the pharmacokinetics of vasopressors given by infusion versus bolus may affect relative dose requirements. Therefore, the aim of this study was to determine the relative potency of norepinephrine and phenylephrine when given as prophylactic infusions to prevent hypotension during CSE anesthesia for cesarean delivery.

## Methods

### Ethics

Ethical approval for this study (Ethical Committee N° KY-2022-04) was provided by the Ethical Committee NAC of Jiaxing Women and Children’s Hospital of Wenzhou Medical University, Jiaxing City, Zhejiang Province, China (Chairperson Prof Xu Shengfeng) on 5 January 2022. And all participating subjects provided written informed consent. The trial was registered prior to patient enrolment in the Chinese Clinical Trials Registry at https://www.chictr.org.cn/(ChiCTR2200056237, principal investigator: Qian Jing, date of registration: 10 January 2022). This clinical trial was from 14 February to 20 march 2022.

### Design

This was a randomized, double-blind, dose finding study using up-and-down allocation method.

### Patients and Setting

Sixty Society of Anesthesiologists (ASA) physical status II women with singleton pregnancy at term (≥37 weeks’ gestation), aged from 20 to 40, scheduled for elective cesarean delivery were enrolled in this study. Exclusion criterion were: body mass index (BMI) > 35 kg/m^2^, height >170 cm or <150 cm, preeclampsia or preexisting hypertension, preexisting or gestational diabetes, fetal distress or intrauterine growth restriction, multiple pregnancy, any contraindications to spinal or epidural anesthesia, including bleeding disorder, local infection or intracranial hypertension.

Patients were randomly allocated to either the norepinephrine group or the phenylephrine group based on computer-generated random numbers (Microsoft Excel, Redmond, WA) by a fixed study assistant. Then the randomized scheme was kept in sequentially numbered opaque envelopes of which one was opened for each patient enrolled.

### Study Protocol

All patients fasting for solid food >6 h and light beverages >2 h. No premedication was given. On arriving in the operating theater, standard monitoring including non-invasive blood pressure, pulse oximetry, and electrocardiogram was applied and an 18-gauge cannula was inserted into a forearm vein. No prehydration was given. Values for baseline systolic blood pressure (SBP) and heart rate (HR) were recorded as the mean of three readings with a difference of <10% measured at 3-min intervals while the patient was resting.

We administered CSE anesthesia at the L3-4 vertebral interspace (assessed by ultrasound) with the patient in the left lateral position using a needle-through-needle technique. The epidural space was located with an 18-gauge Tuohy needle using a loss-of-resistance to saline technique. A 25-gauge Whitacre needle was then inserted through the Tuohy needle and after confirming free flow of cerebrospinal fluid (CSF), hyperbaric bupivacaine 10 mg and sufentanil 5 μg was injected intrathecally at a speed of 0.1 ml/s. Before withdrawing the Whitacre needle gentle aspiration was performed; if CSF could not be identified the case was excluded from the study. A 19-gauge nylon epidural catheter with a single terminal hole was then inserted 3, 4 cm into the epidural space. The catheter was then gently aspirated to exclude the presence of CSF or blood and was fixed with a dressing. The patient was then returned to the tilted supine position.

The study vasopressor solutions were prepared in unmarked 50 ml syringes prior to commencement of each case by an anesthesia assistant who diluted norepinephrine with saline to a concentration of 8 μg/ml or phenylephrine to a concentration of 100 μg/mL. A dedicated anesthesiologist who was not involved in data collection operated the vasopressor infusion pump with the aim of maintaining the SBP in the range of ±20% of baseline. After completion of intrathecal injection, the prophylactic vasopressor infusion was started at the rate determined by the research schedule. Concurrently, an infusion was commenced of 10 ml/kgh warmed lactated Ringer’s solution over 20–30 min, and then patients received a baseline infusion of 500–600 ml warmed lactated Ringer’s solution. The vasopressor infusion pump was stopped if SBP increased to ≥120% of the baseline value and was restarted when the SBP returned to <120% of the baseline value. To facilitate blinding to the anesthesiologists (who collect data) and patients, the screen of the infusion pump was obscured with an opaque cover. Hypotension was defined as a decrease in SBP to ≥20% of the baseline value or <90 mm Hg and was treated with an intravenous bolus of phenylephrine 50 μg. Hypertension was defined as an increase in SBP to ≥120% of the baseline value and was managed by stopping the vasopressor infusion. Bradycardia was defined as a decrease in HR to <50 beats/min. Bradycardia without hypotension was managed by stopping the vasopressor infusion and restarting when the HR increased to >50 beats/min. Bradycardia with hypotension was treated with an intravenous bolus of atropine 0.5 mg and/or ephedrine 6 mg.

According to previous studies (Xiao et al., 2020a; Xu et al., 2021), the first patient in the norepinephrine group received an infusion at 0.1 μg/kg/min and the first patient in the phenylephrine group received an infusion at 0.5 μg/kg/min. The infusion rate for each subsequent patient was adjusted according to up-and-down sequential allocation methodology (Dixon, 1991; Xiao et al., 2020b). An effective infusion rate was defined when no hypotension occurred during the period from induction of anesthesia to delivery. If the infusion was effective, the infusion rate for the next patient was decreased by either 0.1 μg/kg/min in the norepinephrine group or 0.05 μg/kg/min in the phenylephrine group. If the infusion was not effective, the infusion rate for the next patient was increased by the same magnitude.

The sensory block level was measured at 5-min intervals after intrathecal injection by assessing loss of pinprick sensation using the tip of an 18-gauge epidural needle. Surgery was not permitted to start if the sensory block level not reached the T6 dermatome. The SBP and HR were recorded at 1-min intervals until delivery, then at 5-min intervals until the completion of surgery.

### Measurements

The primary outcome of this study was whether the infusion rate was effective or not. Secondary outcomes included the incidences of hypotension, hypertension, bradycardia, nausea and vomiting, and shivering, and neonatal outcome assessed by 1-min and 5-min Apgar scores and umbilical arterial pH. We also recorded the induction-to-delivery intervals and the duration of surgery.

### Sample Size Estimation

The sample size of 30 patients for each group was determined according to previous recommendations that 20–40 subjects would provide a stable estimation of parameters when using the up-and-down allocation methodology for most realistic scenarios (Pace and Stylianou, 2007).

### Statistical Analysis

The demographic characteristics, surgery data and sensory block level were presented using descriptive statistics. Normality of distribution of continuous variables was assessed using the D'Agostino-Pearson omnibus normality test. Normally distributed data were presented as mean ± SD and analyzed with Student’s t test. Non-normally distributed data were presented as median (range) and analyzed with the Mann-Whitney U test. Categorical data were presented as number (%), and analyzed using the chi-square test. The infusion rate that was effective in 50% of patients (ED50) was estimated by calculating the mean of the midpoints of pairs of infusion rate in successive parturients in which an ineffective response was followed by an effective response (crossover) based on modified up-and-methodology as previously described (Dixon, 1991; Xiao et al., 2020b), with 95% confidence interval (CI) and standard error for ED50 values estimated using the method described by Choi (Fieller, 1940; Choi, 1990). The potency ratio with 95% CIs for norepinephrine:phenylephrine was estimated by calculating the ratio of the ED50 values using Fieller’s method (Pace and Stylianou, 2007). Probit regression was used as a backup and sensitivity analysis to estimate ED50 and ED90 values.

Analyses were performed using GraphPad Prism version 5.0 (GraphPad Software Inc., San Diego, CA) and IBM SPSS Statistics for Windows version 22.0 (IBM Corp, Armonk, NY). Values of *p* < 0.05 were considered statistically significant.

## Results

**FIGURE 1:**
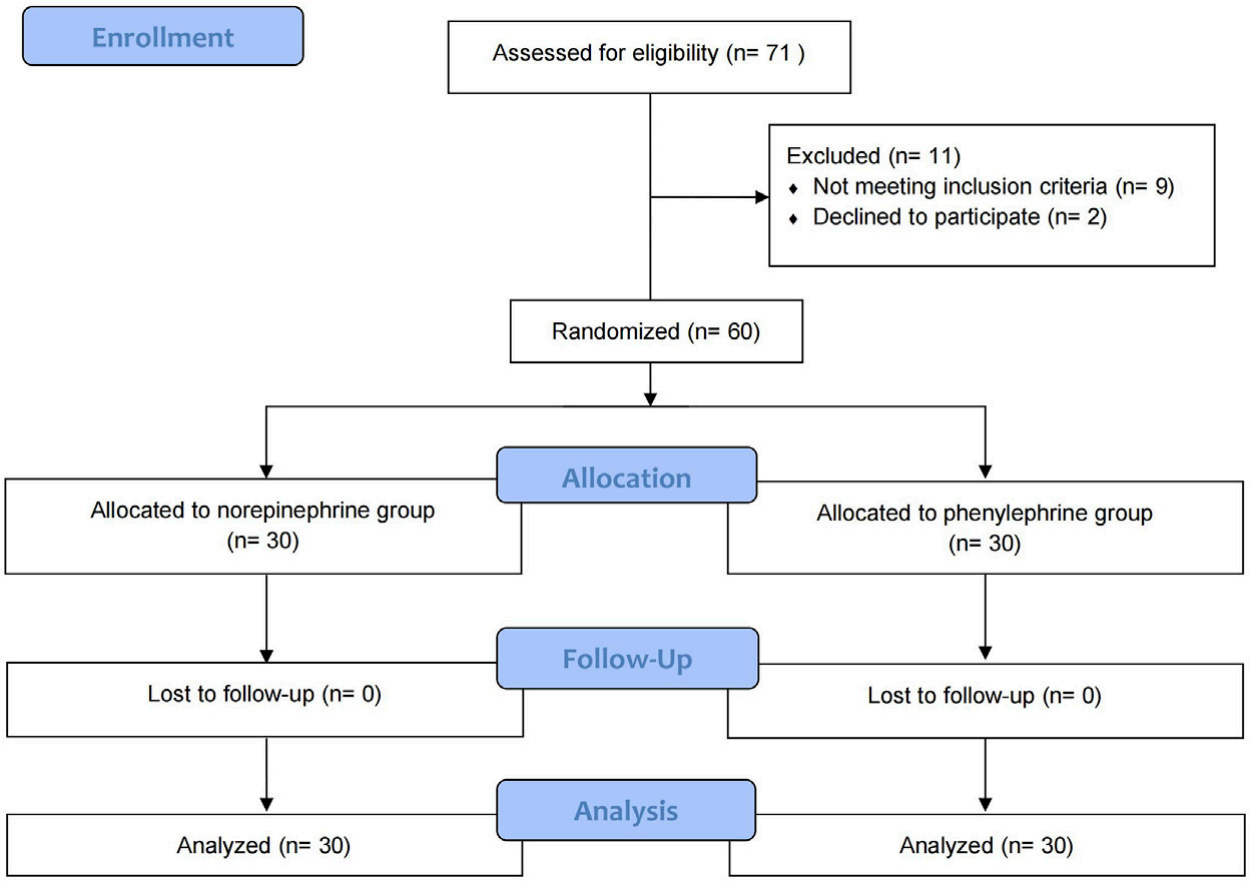
CONSORT diagram showing patient recruitment and flow.

Patient flow is shown in Figure 1. Seventy-one patients were assessed for eligibility of whom 60 were randomly allocated into the two groups and were included in the final analysis. Patient demographics, surgical times and time to maximum sensory block are shown in Table 1.

**TABLE 1:** Patient characteristics and surgical times.

	Phenylephrine Group (*n* = 30)	Norepinephrine Group (*n* = 30)	*p* Value
Age (yr)	31.8 ± 3.9	32.0 ± 3.5	0.86
Height (cm)	158.5 ± 4.9	159.3 ± 4.1	0.51
Body mass index (kg/m^2^)	29.0 ± 2.5	26.9 ± 3.0	0.05
Gestational age (wk)	38.7 ± 0.8	39.0 ± 0.8	0.21
Time from spinal induction to delivery (min)	15.7 ± 3.4	15.8 ± 3.6	0.97
Duration of surgery (min)	45.5 (37.0–55.0)	51.0 (42.8–58.5)	0.16
Time to maximum sensory block (min)	6.0 (6.0–8.0)	7.0 (6.8–7.3)	0.49
Maximum sensory block	T4 (T4 - T4)	T4 (T4 - T4)	0.60
Amount of bleeding (ml)	400 (350.0-450.0)	395 (300.0-442.5)	0.34
Urine volume (ml)	175 (100.0-200.0)	150 (100.0-200.0)	0.85
Intravenous crystalloid during study period (ml)	193.8 ± 42.0	184.5 ± 52.6	0.45

Data are mean ± SD or median (interquartile range).

**FIGURE 2:**
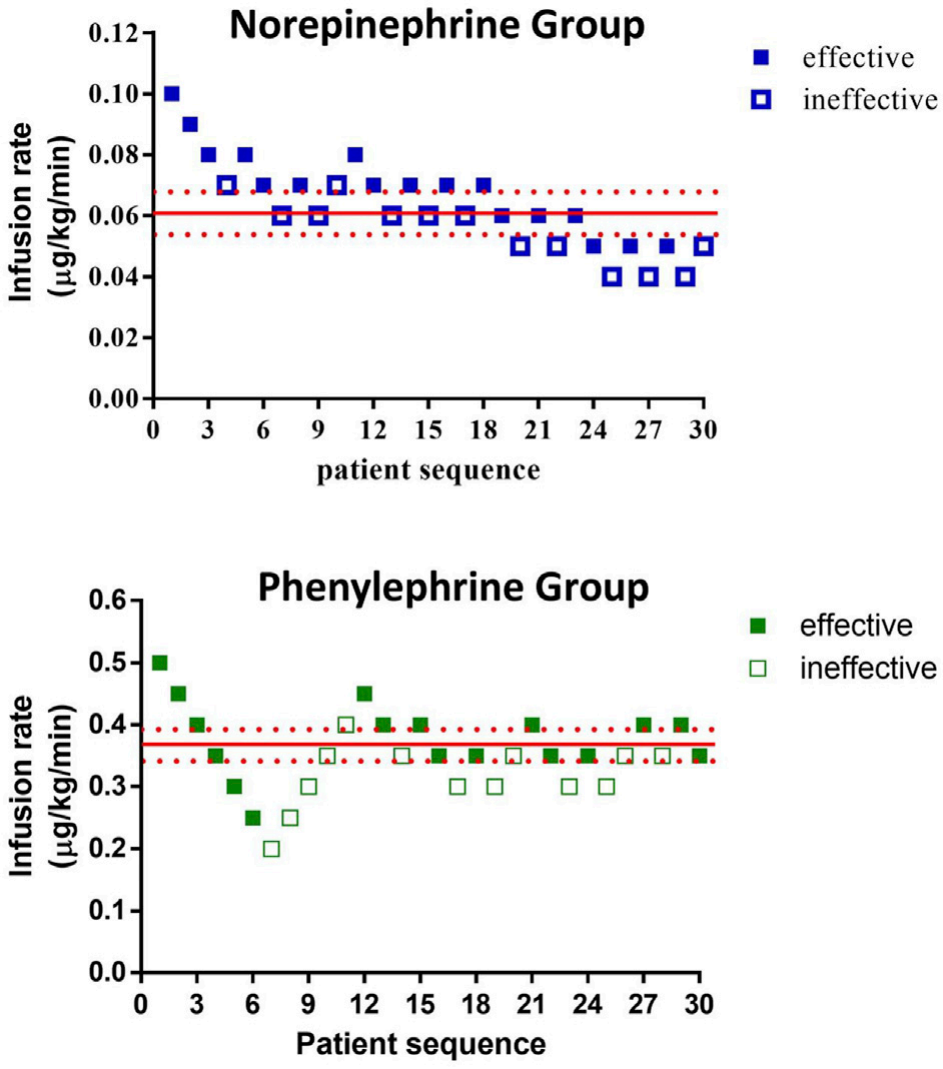
Observed proportions of subjects with effective or ineffective responses to different infusion rates of norepinephrine or phenylephrine.

**FIGURE 3:**
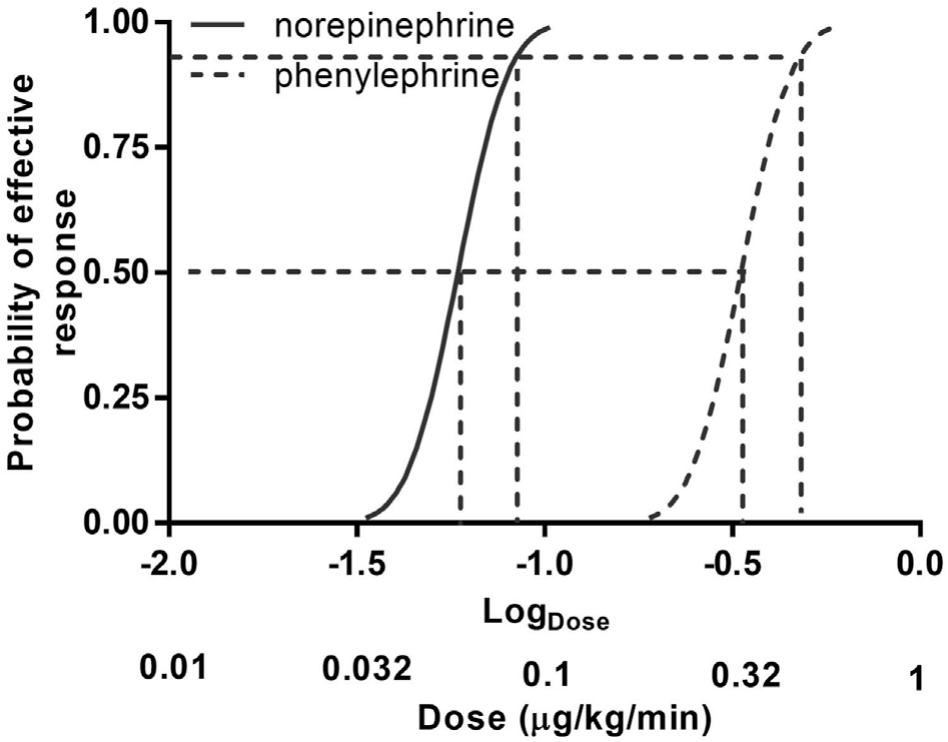
Dose–response curves of phenylephrine and norepinephrine infusions for preventing hypotension calculated using probit analysis. Values for dose represent infusion rates. The horizontal axis is on a logarithmic scale. Antilog values for dose are shown below log (dose) values to aid interpretation.

The sequences of patients are shown in Figure 2. The estimated value for ED50 was 0.061 (95% CI 0.054–0.068) μg/kg/min for norepinephrine and 0.368 (95% CI 0.343–0.393) μg/kg/min for phenylephrine. The estimated relative potency ratio for ED50 for norepinephrine:phenylephrine was 6.03:1 (95% CI 5.26:1 to 6.98:1). The dose-response curves generated using probit regression are shown in Figure 3. The estimated values for ED50 and ED90 estimated by probit regression were 0.059 (95% CI 0.048–0.068) μg/kg/min and 0.080 (95% CI 0.069–0.120) μg/kg/min respectively for norepinephrine and 0.330 (95% CI 0.278–0.376) μg/kg/min and 0.449 (95% CI 0.390–0.679) μg/kg/min respectively for phenylephrine. The estimated relative potency ratio for ED90 for norepinephrine:phenylephrine was 5.68:1 (95% CI 5.16:1 to 6.75:1).

The incidence of side effects and neonatal outcome are shown in Table 2; there were no differences between groups.

**TABLE 2:** Side effects and neonatal outcome.

	Phenylephrine Group (*n* = 30)	Norepinephrine Group (*n* = 30)	*p* Value
Side effects			
Hypotension	12 (40%)	14 (46.7%)	0.60
Hypertension	2 (6.7%)	0 (0%)	0.47
Bradycardia	0 (0%)	0 (0%)	—
Nausea/vomiting	5 (16.7%)	4 (13.3%)	1.0
Shivering	12 (40%)	10 (33.3%)	0.59
Neonatal outcome			
Neonatal weight (g)	3,394 ± 385	3,377 ± 369	0.87
Apgar score (1 min)	10.00 (9.75-10.00)	10.00 (9.00-10.00)	0.52
Apgar score (5 min)	10.00 (10.00-10.00)	10.00 (10.00-10.00)	0.49
Umbilical arterial pH	7.31 ± 0.04	7.33 ± 0.05	0.13

Data are number (%), mean ± SD, or median (interquartile range).

## Discussion

In this randomized double-blinded study, the relative dose requirements for norepinephrine and phenylephrine administered as fixed-dose continuous infusions to prevent hypotension during CSE anesthesia for elective cesarean delivery were determined. The estimated relative potency for norepinephrine:phenylephrine was about 6:1. This means, for example, that an infusion rate of norepinephrine 0.1 μg/kg/min would be equivalent to an infusion rate of phenylephrine 0.6 μg/kg/min.

Previous studies of the relative potency of norepinephrine and phenylephrine during spinal anesthesia for cesarean delivery have investigated administration by bolus. In a random-allocation graded dose response study, Ngan Kee et al. investigated norepinephrine and phenylephrine given a single bolus to treat the first episode of hypotension and estimated the relative potency for norepinephrine:phenylephrine as 13.1 (95% CI 10.4–15.8) (Ngan Kee, 2017b). Mohta et al. also compared single boluses of norepinephrine and phenylephrine to treat hypotension (Mohta et al., 2019). Using up-and-down sequential allocation methodology they estimated the relative potency for norepinephrine:phenylephrine as 11.3 (95% CI 8,1–16.9). The estimate of relative potency of norepinephrine:phenylephrine from our study is lower that found in the previous two studies (Ngan Kee, 2017b; Mohta et al., 2019). This may be explained by differences in the methodology between studies and may reflect differences in the relative pharmacokinetics of administration of vasopressors by bolus and infusion. Comparison of our results with those of the previous studies suggests that the dose requirement for norepinephrine relative to phenylephrine is greater when the vasopressors are given by infusion compared with bolus. The reason for this is unclear but might be explained if the duration of action of norepinephrine is shorter than that of phenylephrine, thus requiring a greater cumulative dose when given by infusion. However, further work is required to confirm this.

In clinical obstetric anesthesia practice, administration of vasopressors by infusion has been advocated to prevent hypotension during spinal anesthesia and it has been suggested that this is superior to administration by bolus for reducing the incidence of hypotension and associated nausea and vomiting (Kinsella et al., 2018; Singh et al., 2020). Although several previous studies have recommended the use of infusions of norepinephrine, recommendations for rate of infusion have varied, ranging from 0.05 to 0.1 μg/kg/min (Hasanin et al., 2019a; Fu et al., 2020; Wei et al., 2020; Xu et al., 2021). Most units are likely to be more experienced with the use of infusions of phenylephrine and may have developed guidelines for its use. The results of our study may provide guidance for these units for the appropriate infusion rate of norepinephrine based on their current phenylephrine regimen.

Recently, increasing studies have reported about the infusion of phenylephrine and norepinephrine managing the hemodynamics during cesarean delivery under spinal anesthesia. (Ngan Kee et al., 2015b) firstly compared the two vasopressor administration via computer-controlled infusion, and found norepinephrine was superior in maintenance of patients’ heart rate and cardiac output when compared with phenylephrine. (Hasanin et al., 2019b) compared phenylephrine 0.75 μg/kg/min and norepinephrine 0.05 μg/kg/min for prophylaxis against post-spinal anaesthesia hypotension during elective cesarean delivery. They found maintaining maternal SBP with norepinephrine infusion was associated with less number of physician interventions and less incidence of bradycardia and reactive hypertension when comparing with phenylephrine infusion. (Ngan Kee et al., 2020b) conducted a non-inferiority study to compare phenylephrine and norepinephrine; they found the latter was non-inferior to the former for neonatal outcome evaluated using umbilical arterial pH. These encouraging studies indicate that norepinephrine has the potential to replace norepinephrine as a first-line drug in obstetric anesthesia.

It should be noted that the ED50 for norepinephrine in this study is different from the values of previous studies reported (Fu et al., 2020; Wei et al., 2020). Different methodologies were used in these studies may be account for the inconsistency. In our studies, we chose up-and-down allocation method to determine the ED50 by calculating the mean of the midpoints of pairs of infusion rate in successive parturients in which an ineffective response was followed by an effective response (crossover) based on modified up-and-methodology as previously described (Dixon, 1991; Pace and Stylianou, 2007). Otherwise, (Fu et al., 2020) and (Wei et al., 2020) used the randomized dose allocation method and calculated the ED50 and ED90 by probit regression. Fortunately, the primary aim of this study is to determine the relative potency ratio of the two vasopressors, which may be not influenced by methodology.

There may be concern among some obstetric anesthesiologists about the safety of peripheral intravenous norepinephrine infusion (Smiley, 2017). However, the risk of norepinephrine infusion should be similar to that of phenylephrine infusion when the drugs are given in equipotent dilute concentrations (Ngan Kee et al., 2018). This is supported by the results of a large multicenter study of non-obstetric patients that found no significant association between the use of peripheral norepinephrine infusions and adverse events (Pancaro et al., 2020). Nevertheless, we recommend peripheral administration of norepinephrine via a large vein with a concurrent intravenous fluid infusion through the same vein.

Our study has a number of limitations. First, the up-down sequential allocation method used was focused on the estimation of the value of ED50. We used probit regression as a backup and sensitivity analysis. Using the latter, we also estimated ED90 values for norepinephrine and phenylephrine. Because the latter values were obtained by extrapolation and the estimated values had wide 95% CIs, these results should be viewed only as approximations. Second, we included only healthy patients having elective surgery. Our results may not be generalizable to patients with concurrent disease or having non-elective surgery.

In summary,under the conditions of this study, norepinephrine given by infusion was about 6 times more potent than phenylephrine. This information is useful for clinical practice and further comparative studies of norepinephrine versus phenylephrine.

## Data Availability

The raw data supporting the conclusion of this article will be made available by the authors, without undue reservation.
